# Immune Checkpoint Inhibitors in Advanced NSCLC: [^18^F]FDG PET/CT as a Troubleshooter in Treatment Response

**DOI:** 10.3390/diagnostics11091681

**Published:** 2021-09-15

**Authors:** Cristina Ferrari, Giulia Santo, Nunzio Merenda, Alessia Branca, Paolo Mammucci, Pamela Pizzutilo, Cosmo Damiano Gadaleta, Giuseppe Rubini

**Affiliations:** 1 Nuclear Medicine Unit, Interdisciplinary Department of Medicine, University of Bari Aldo Moro, Piazza Giulio Cesare 11, 70124 Bari, Italy; giuliasanto92@gmail.com (G.S.); nu.me@hotmail.it (N.M.); alessia9130@gmail.com (A.B.); paolo.mammucci@outlook.com (P.M.); giuseppe.rubini@uniba.it (G.R.); 2 Medical Thoracic Unit, IRCCS Oncologico Giovanni Paolo II, 70124 Bari, Italy; pamela.pizzutilo@gmail.com; 3 Interventional and Medical Oncology Unit, IRCCS Istituto Tumori “Giovanni Paolo II”, Viale Orazio Flacco 65, 70124 Bari, Italy; c.gadaleta@oncologico.bari.it

**Keywords:** immunotherapy, PD-1, NSCLC, [^18^F]FDG PET/CT, treatment response, IrAEs

## Abstract

Introduction: The aim of this study was to investigate whether [^18^F]FDG PET/CT-derived semi-quantitative parameters can predict immunotherapy treatment response in non-small cell lung cancer (NSCLC) patients. Secondly, immune-related adverse events (irAEs) and lymphoid cell-rich organs activation were evaluated. Materials and Methods: Twenty-eight patients who underwent [^18^F]FDG PET/CT scans before and at first restaging therapy with immuno-checkpoint inhibitors (ICIs) were retrospectively analyzed. PET-based semi-quantitative parameters extracted from both scans were respectively: SUV_max_ and SUV_peak_ of the target lesion, whole-body metabolic tumor volume (MTV_WB_), and whole-body total lesion glycolysis (TLG_WB_), as well as their interval changes (ΔSUV_maxTL_, ΔSUV_peakTL_, ΔMTV_WB_, ΔTLG_WB_). These PET-derived parameters were correlated to controlled disease (CD) assessed by RECIST 1.1. IrAEs, if present, were also described and correlated with clinical benefit (CB). SUV_max_ of the spleen and bone marrow at restaging scans were also correlated to CB. Results: The CD was achieved in 54% of patients. Out of 28 eligible patients, 13 (46%) experienced progressive disease (PD), 7 showed SD, 7 had PR, and only in one patient CR was achieved. ΔSUV_maxTL_ (*p* = 0.002) and ΔSUV_peakTL_ (*p* < 0.001) as well as ΔMTV_WB_ (*p* < 0.001) and ΔTLG_WB_ (*p* < 0.005) were significantly associated with PD vs. non-PD. IrAEs and lymphoid cell-rich organs activation did not correlate with CB. Conclusions: [^18^F]FDG PET/CT by using interval changes of PET-derived semi-quantitative parameters could represent a reliable tool in immunotherapy treatment response evaluation in NSCLC patients.

## 1. Introduction

In the last decade, the advent of immunotherapy has paved the way for new treatment options for patients with advanced non-small cell lung cancer (NSCLC) [1]. Immuno-checkpoint inhibitors (ICIs) therapy exploits the use of antibodies that target specific molecules involved in tumor signaling, determining the suppression of cytotoxic T lymphocytes [2]. In addition, by releasing the brakes of the host-immune system, ICIs may alter the physiological homeostasis of immune response, thus leading to the development of immune-related adverse events (irAEs) [3]. To date, a standard method to evaluate the success of these innovative therapies and to identify patients who may benefit from them remains undetermined. Morphological imaging by using Response Evaluation Criteria in Solid Tumors (RECIST1.1) represents the standard modality to cytotoxic therapies response assessment [4]. To face this new clinical issue, the immune-related Response Evaluation Criteria in Solid Tumor (irRECIST) was developed [5], but their efficacy in early treatment response evaluation is still limited [6].

Fluorine-18 fluorodeoxyglucose positron emission tomography/computed tomography ([^18^F]FDG PET/CT) represents an essential diagnostic tool in the management of NSCLC patients, from staging to treatment response evaluation. Even in the new scenario of immunotherapy, [^18^F]FDG PET/CT, thanks to the semi-quantitative analysis and PET-derived parameters, could represent a reliable diagnostic technique offering additional information to standard modalities [7,8]. Several studies investigated the role of PET extracting data before and/or during immunotherapy [9]. Among them, standardized uptake value (SUV), the most commonly used, was reported to be correlated to response rate. Takada et al., in 89 patients with advanced or recurrent NSCLC, showed that patients with a baseline SUVmax ≥ 11.16 had a significantly higher response rate compared to patients with lower SUVmax values [10]. A plethora of evidence is already available on the predictive role of volume-based PET-parameters. Some authors highlighted the predictive value of metabolic tumor volume (MTV) as well as the total lesion glycolysis (TLG) in NSCLC patients treated with ICIs [11]. Despite the increased amount of evidence, a common agreement has still not been reached.

Interestingly, [^18^F]FDG PET/CT could reveal irAEs before their clinical manifestation and/or laboratory test positivity. However, few literature data are still available regarding the irAEs imaging features and radiological description. Consequently, the relation between irAEs and treatment response needs further investigation [12].

This study aims to evaluate the role of [^18^F]FDG PET/CT-derived semi-quantitative parameters in the immunotherapy response assessment. Secondly, the detection of immune-related adverse events (irAEs) and lymphoid cell-rich organs activation were investigated in order to evaluate their possible predictive value.

## 2. Materials and Methods

### 2.1. Subject

A single-center database was retrospectively interrogated to identify patients with a history of advanced NSCLC treated with ICIs who underwent [^18^F]FDG PET/CT. The inclusion criteria were: (a) histologically/cytologically proven NSCLC; (b) [^18^F]FDG PET/CT scans before and at first restaging after the start of immunotherapy; (c) minimum follow-up of 3 months after treatment initiation; (d) radiological assessment during treatment every 8–12 weeks with CT scans, for good clinical practice; and (e) the availability of information on the best clinical response to immunotherapy.

All patients were observed for at least 6 months after the first restaging [^18^F]FDG PET/CT, except for those who died. Age, sex, histological subtypes, previous surgery, prior lines, and type of therapy and molecular profile (if available) were also collected.

All patients had already given their consent for the use of their data for clinical research. Our Institutional Review Board does not require the Ethical Committee’s approval for review of patients’ files.

### 2.2. [^18^F]FDG PET/CT Examination and Analysis

[^18^F]FDG PET/CT scans were performed with a Discovery 710 PET/CT scanner (GE, Healthcare Technologies, Milwaukee, WI, USA), and the same scanner was used for baseline and first post-treatment evaluation. All patients fasted for at least 6 h and presented a blood glucose levels less than 200 mg/dL. An intravenous injection of 3.0 MBq/kg of [^18^F]FDG was administered and PET/CT scanning was performed 60 min after injection. Non-contrast CT images and subsequent PET images were acquired from the skull base to the upper thigh in the supine position with the arms raised. PET image acquisition was performed for 4 min per bed in 3-dimensional acquisition mode using 7 to 10 beds. Image review and analysis were conducted on a dedicated workstations and software (AW Server 4.7; GE, Healthcare Technologies, Milwaukee, WI, USA).

All PET/CT scans were reviewed by a nuclear medicine expert who performed visual interpretation and semiquantitative analysis, documenting all pathological foci of FDG uptake as well as the appearance of abnormal metabolism (non-physiologic) in organs possibly activated by immune-system response at first restaging PET/CT images.

Over PET parameters, the standardized uptake value (SUV) was evaluated in terms of SUV_max_ and SUV_peak_. In addition, volume-based PET parameters, MTV and TLG were obtained and used for further analysis.

PET-based semi-quantitative parameters extracted from both scans were respectively: SUV_max_ and SUV_peak_ of the target lesion (preSUV_maxTL_, postSUV_maxTL_, preSUV_peakTL_, postSUV_peakTL_), whole-body MTV (preMTV_WB_ and postMTV_WB_), and whole-body TLG (preTLG_WB_ and postTLG_WB_), as well as their interval changes (ΔSUV_maxTL_, ΔSUV_peakTL_, ΔMTV_WB_ and ΔTLG_WB_). Moreover, SUV_max_ of lymphoid cell-rich organs, spleen (pre-/post-ΔSUV_maxSp_) and bone marrow (pre-/post-ΔSUV_maxBm_), were collected.

### 2.3. Response Evaluation

The diagnostic assessment was performed according to Response Evaluation Criteria for Solid Tumors criteria version 1.1 (RECIST1.1), in terms of complete response (CR), partial response (PR), stable disease (SD), and progressive disease (PD) at first radiological restaging. The controlled disease (CD) was defined as the achievement of CR, PR, and SD. All forementioned PET-derived parameters were correlated to treatment response.

Moreover, clinical benefit (CB) was determined considering patients’ clinical course after the last follow-up: (a) continued/stopped immunotherapy, (b) disease control/exacerbation, (c) changing to chemotherapy, or (d) death. IrAEs, if present, were also described and correlated, together with SUV_max_ of lymphoid cell-rich organs, to CB at follow up.

### 2.4. Statistical Analysis

Categorical variables were described using absolute and relative frequencies; continuous variables were described using median range. To assess the correlation between each PET/CT parameters and response groups (PD vs. no-PD), the Mann–Whitney test was used, and the results were represented graphically by box-plots. The multivariate Cox model was adopted to assess the association of PET-parameters, adjusting for gender, drugs, line of therapy, and previous lung surgery, both for CD and CB.

Finally, the association between the presence of irAEs at first restaging [^18^F]FDG PET/CT exams and CD as well as CB, was investigated using Chi-square and Fisher’s exact test. A *p* value less than 0.05 was considered statistically significant. All statistical analysis was performed using IBM SPSS Statistic Version 28 (IBM, Armonk, NY, USA).

## 3. Results

Between March 2016 and September 2020, a total of 47 patients with advanced NSCLC performed 103 [^18^F]FDG PET/CT exams in our Nuclear Medicine Department for ICIs treatment response evaluation. Among them, 28 patients met the inclusion criteria and were eligible for the study. Six (21%) patients were female and 22 (79%) were male. Histology revealed adenocarcinoma (79%) as the most common histotype. PD-L1 expression was available in 10/28 patients (5/10: PD-L1 expression < 50%; 5/10 PD-L1 expression > 50%). Patients were equally treated with pembrolizumab and nivolumab (13:15). Table 1 lists of all the patients’ characteristics.

The median time between the baseline PET/CT exam and the start of immunotherapy was 38 days (range: 2–90 days), whereas the median time between PET/CT scans was 4 months (range: 2–11 months).

At baseline, PET/CT was positive in all patients, showing the presence of [^18^F]FDG uptake in lung alone (*n* = 5), extra-lung sites (i.e., local or distant lymph nodes, bone, adrenal glands, *n* = 3), and lung + extra-lung sites (*n* = 20).

**Figure 1:**
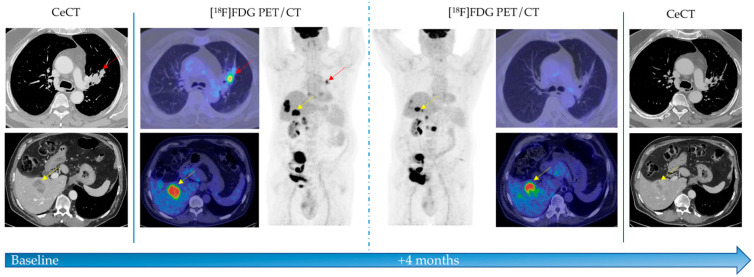
A 60-year old man affected by non-small cell lung cancer underwent [^18^F]FDG PET/CT before and after immunotherapy. PET/CT showed high [^18^F]FDG uptake into primary lesion in the left lung (red arrows) and in the liver (yellow arrows), as secondary disease localizations. After four pembrolizumab cycles, patient underwent morphological and functional revaluation. [^18^F]FDG PET/CT showed no more uptake in primary tumor and a reduction of metabolic activity in liver lesions. CeCT evaluation confirmed partial response (PR) to immunotherapy. CeCT: contrast enhanced computed tomography; [^18^F]FDG PET: Fluorine-18 fluorodeoxyglucose positron emission tomography.

Thirteen (46%) eligible patients, experienced progressive disease (PD) at first restaging, 7 (25%) patients showed SD, 7 (25%) had PR, and only in one patient (4%) CR was obtained. The CD was achieved in 15/28 (54%) patients (Figure 1).

**Figure 2:**
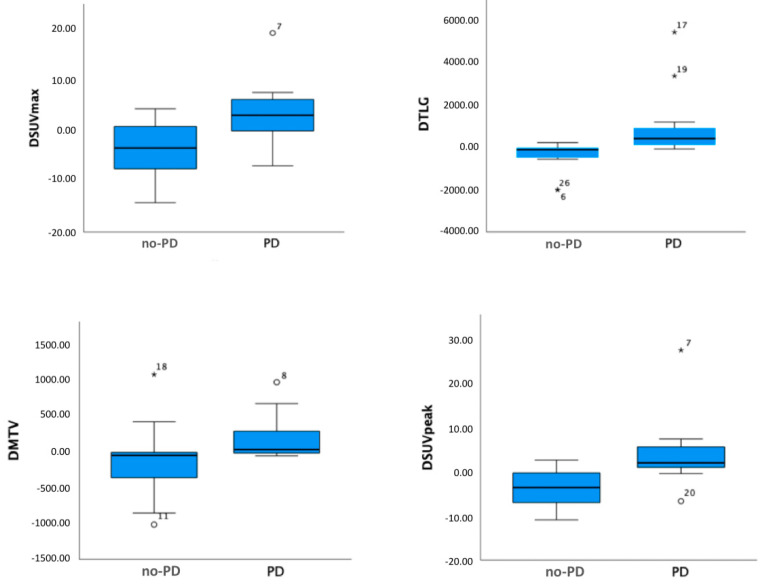
Box plots of PET-derived parameters which significantly correlated with progressive (PD) vs. non-progressive (no-PD) disease. CR: complete response; PR: partial response; SD: stable disease; PD: progressive disease; SUV: standardized uptake value; TLG: total lesion glycolysis; MTV: metabolic tumor volume.The midline, box edges and outers bars indicate the median, first and third quartiles, and the upper and lower whiskers, respectively. Dots (°) and asterisks (*) represent outliers.

None of parameters extracted from the PET/CT before starting immunotherapy (preSUV_maxTL_, preSUV_peakTL_, preMTV_WB_, preTLG_WB_) showed a significant correlation with radiological response. Conversely, ΔSUV_maxTL_ (*p* = 0.002) and ΔSUV_peakTL_ (*p* < 0.001) as well as ΔMTV_WB_ (*p* < 0.001) and ΔTLG_WB_ (*p* < 0.005) were significantly associated with PD vs. non-PD. No difference was showed between the pembrolizumab and nivolumab treated-groups. In the subgroup with available PD-L1 status, the ligand expression was shown to be statistically correlated with baseline SUV_max_ (*p* = 0.017) and baseline SUV_peak_ (*p* = 0.03). The relation between CD and ΔSUV_maxTL_, ΔSUV_peakTL_, ΔMTV_WB_, ΔTLG_WB_ is reported in the box plots in Figure 2.

The median follow-up was 11 months (range: 4–48). Among all patients, 18 (64%) experienced CB: all of them continued immunotherapy and were alive at last follow-up. Conversely, 10 patients (36%) showed no-CB. Among them, 8 (80%) had disease exacerbation and stopped immunotherapy, while 2 (20%) died soon after the first restaging.

**Figure 3:**
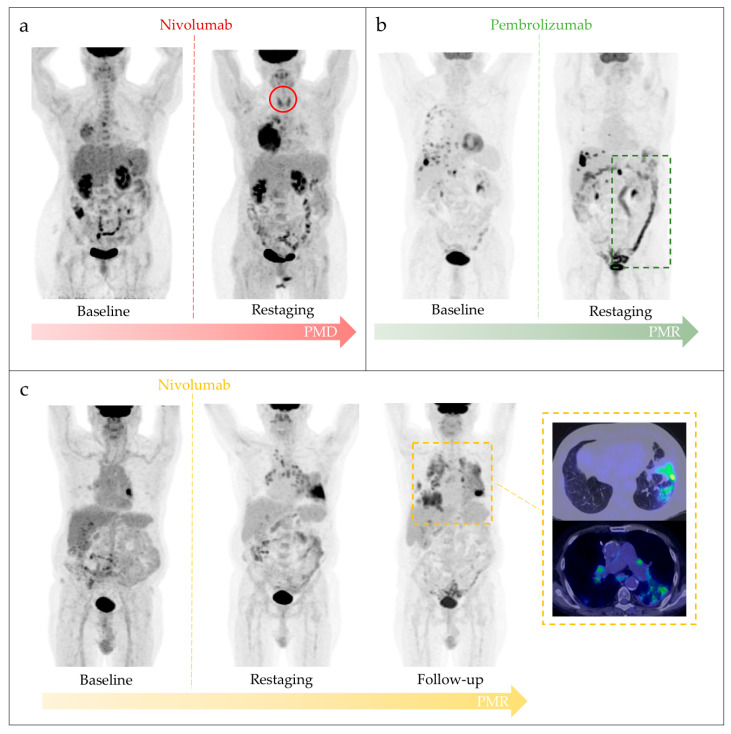
Most representative cases of irAEs detected on [^18^F]FDG PET/CT in our sample. (**a**) Immunotherapy-related thyroiditis: 58yo female with advanced lung adenocarcinoma under nivolumab immunotherapy. After 8 cycles of therapy, asymptomatic thyroiditis was incidentally found on restaging PET/CT (red circle). The same scan showed progressive metabolic disease (PMD), confirmed by follow-up. (**b**) Immune-related colitis: 64yo man affected by stage IV NSCLC, with pleural involvement and lymph nodes metastasis detected on basal PET/CT. During pembrolizumab treatment, the patient reported persistent diarrhea. Restaging PET/CT scan showed partial metabolic response (PMR) and increased [^18^F]FDG uptake in the colon region consistent with immune-mediated colitis (green dotted square). (**c**) Immune-related pneumonitis and sarcoid-like reaction: 56yo man with stage IIIA NSCLC who received nivolumab monotherapy as first line treatment. Basal PET/CT showed [^18^F]FDG uptake both in the left pulmonary lesion and hilar bilateral lymphadenopathies. First restaging [^18^F]FDG PET/CT showed increase in bilateral mediastinal and hilar lymphadenopathy involvement and diffuse lung parenchymal uptake, suggesting a sarcoid-like reaction together with a pneumonitis (yellow dotted square), the latter supported by the presence of dyspnea and dry cough. Nivolumab was continued and subsequent PET/CT scan showed a partial metabolic response (PMR).

Five patients showed immuno-related findings on PET/CT. Notably, in two out of the five patients, diffuse thyroid [^18^F]FDG uptake, as for thyroiditis, was detected. PET/CT finding of colitis was observed in one patient only, who reported persistent diarrhea during pembrolizumab treatment. A pattern of immunotherapy-related arthritis and pneumonitis associated with sarcoid reaction was shown on PET/CT scans of the last two patients, respectively. Table 2 shows the demographics of patients who developed irAEs and the most representative cases are depicted in Figure 3.

Three out of the five patients with irAEs PET/CT findings reached CD (1 SD; 2 PR), while the other two showed PD on morphological imaging. No statistically significant correlation was reached between irAEs and CD (*p* = 0.429). Similarly, three out of five patients showed CB at follow-up but no significant correlation was found (*p* = 0.229).

None of lymphoid cell-rich organs metabolic PET-parameters was significantly correlated to CB. All statistical results are detailed in Table 3 and Table 4.

## 4. Discussion

The advent of immunotherapy in clinical practice and the approval of several drugs for advanced solid tumors led to the development of new challenges for imaging. In this scenario, [^18^F]FDG PET/CT, a consolidate tool in the evaluation of lung cancer, was proposed as a promising marker for immunotherapy treatment response evaluation, providing useful and unique information [13].

In the last few years, there has been a growing interest in the evaluation of PET-derived parameters as predictors of response [14]. This study pointed out that metabolic changes during immunotherapy statistically correlate with treatment response as well as with CB. Notably, the interval changes of all PET-derived parameters showed a significant decrease in responders, potentially representing a marker of long-term clinical response. Similar results were reached by Nobashi et al., in their study on 40 patients affected by different cancer types, where they found a significant correlation between PET parameters’ variation and best overall response at one year [15].

Other authors assessed the predictive role of [^18^F]FDG PET/CT by semiquantitative analysis. In 2019, Evangelista et al. retrospectively studied 32 patients with metastatic lung cancer under nivolumab treatment. SUV_maxWB_ was significantly higher in patients with PD compared with those with SD and PR, whereas a similar trend was shown for TLG_WB_ and MTV_WB_, both higher in non-responders than responders, even without a statistical significance [16]. Conversely, in other studies, SUV_max_ was unable to predict response. However, volume-based PET parameters were extracted. In this context, a bicentric Italian study performed an analysis in a bigger cohort of 92 patients, demonstrating that those with PD had higher MTV median values compared to those with controlled disease [17]. Similarly, Polverani and colleagues observed a significant association of MTV and TLG of the primary lesions with PD, since lower MTV and TLG values were associated with non-PD status [18]. In our study, we emphasized the variation of PET-derived parameters during immunotherapy in NSCLC patients as a potential biomarker of treatment response.

Even if PD-L1 status was not available for all patients included in our sample, the analysis revealed its significant correlation with baseline SUVs: increased values of SUVs were detected in patients with PD-L1 expression higher than 50%. Albeit with a limited number of cases, this finding, supported by the literature data, suggests a potential role of [^18^F]FDG PET/CT in predicting PD-L1 status [19,20].

Interestingly, in the multivariate analysis, it was shown that immunotherapy employed as a second or more line of therapy was significantly correlated to a better response rate. We can speculate that this finding could be associated with the growing evidence that cytotoxic chemotherapy and radiotherapy could impact tumor ligand expression, determining changes in cell PD-L1 expression as well as in the tumor microenvironment [21,22].

There is still a lack of data on the role of irAEs in predicting the response to ICIs. A first 2017 retrospective trial reported data from 134 NSCLC patients treated with nivolumab. A total of 51% of patients had irAEs with a statistically significant longer progression free survival (PFS) and OS [23]. In another prospective trial, 30% of irAEs were found in a total of 38 patients who showed a better objective overall response (ORR) [24]. Though promising, the data are conflicting. The most reported frequent irAEs were endocrinopathy (hypothyroidism 4–8%, hyperthyroidism 0–5%), skin rashes (5–11%), and hepatitis (2–11%). The most severe ones were pneumonitis (3–5%), colitis (1–2%), hypophysitis (2%), and adrenal failure (0–1%), estimated to occur in 69% of patients [25].

Even if the occurrence of irAEs seems to be associated with better response and prolonged overall survival (OS) [26], few data are available in the literature about the prognostic role of irAEs detected on PET and response. In a study conducted by Sachpekidis et al. in a cohort of metastatic melanoma patients, irAEs on PET/CT correlated with response to immunotherapy—patients who developed at least one irAE had a significantly longer PFS than those without irAEs [27,28].

In our sample, any statistically significant correlation was reached, probably due to the few immune-related events frequency registered. However, three out of five patients who showed irAE on PET/CT experimented CD or CB. Moreover, as PET/CT could monitor the metabolic changes in peripheral lymphoid organs and related ones [29], we conducted a semi-quantitative evaluation on lymphoid cell-rich organs, such as the spleen and bone marrow but none of PET-derived parameters were shown to be statistically significant. Conversely, Nobashy et al. conducted a similar analysis reporting a significant increase of SUV_max_ of the spleen in those patients who did not show any clinical benefit after the start of immunotherapy [15].

Despite the retrospective nature of the present study and the small population size that could impact the results, our study aimed to help highlight the emerging and promising role of PET imaging in predicting response in the field of immunotherapy.

## 5. Conclusions

[^18^F]FDG PET/CT could represent a reliable and efficacious diagnostic tool in immunotherapy treatment response evaluation in advanced NSCLC patients. The decrease of all tumor parameters at first restaging PET/CT results in a predictive role for immunotherapy response and could represent a useful biomarker to estimate treatment response evaluation. Further research is needed to confirm these preliminary data and to explore the interesting field of irAEs findings on PET/CT and their correlation with response.

## Figures and Tables

**Table 1:** Patients’ characteristics.

Variable	Number
Total Number of Patients	28
Median age at diagnosis (years)	65 (range 48–87)
Sex	
Male	22 (79%)
Female	6 (21%)
Histological variant	
Adenocarcinoma	22 (79%)
Squamous Cell Carcinoma	4 (14%)
Others	2 (7%)
Previous lung surgery	
No	21 (75%)
Yes	7 (25%)
Immunotherapy	
First line	8 (29%)
≥Second line	20 (71%)
Drugs	
Nivolumab	15 (54%)
Pembrolizumab	13 (46%)

**Table 2:** Patients’ irAEs.

Patients (*n* = 5)	Age, Sex	Disease	Therapy	irAEs	Final Outcome
1	58, F	NSCLC	Nivolumab	Thyroiditis	PD
2	61, F	NSCLC	Nivolumab	Thyroiditis	SD
3	64, M	NSCLC	Pembrolizumab	Colitis	PR
4	64, M	NSCLC	Nivolumab	Arthritis	PD
5	60, M	NSCLC	Nivolumab	Pneumonitis and sarcoid reaction	PR

F: female; M: male; NSCLC: non-small cell lung cancer; irAEs: immune-related adverse events; PR: partial response; PD: progressive disease; SD: stable disease.

**Table 3:** PET-derived parameters and interval change.

	Overall (*n* = 28)		
CD	**PET Parameters**	**Median ± SD**	***p***
	preSUV_maxTL_	13.0 ± 5.4	0.751
	preSUV_peakTL_	10.0 ± 4.2	0.525
	preTLG_WB_	425,737 ± 586.6	0.130
	preMTV_WB_	203.0 ± 302.9	0.387
	ΔSUV_max TL_	−0.5 ± 6.7	0.003
	ΔSUV_peak TL_	−0.04 ± 7.2	<0.001
	ΔTLG_WB_	242.8 ± 1375.6	<0.001
	ΔMTV_WB_	34.8 ± 443.9	0.022
CB	**Lymphoid Cell-Rich Organs**	**Median ± SD**	***p***
	postSUV_maxSp_	2.3 ± 0.6	0.586
	postSUV_maxBM_	2.0 ± 0.4	0.464

CD: disease control; CB: clinical benefit; SUV: standardized uptake value; TLG: total lesion glycolysis; MTV: metabolic tumor volume; TL: target lesion; WB: whole-body; Sp: spleen; BM: bone marrow.

**Table 4:** Predictive role of patients’ clinical pathological features and PET-derived parameters in multivariate analysis.

Patients (*n* = 28)	Controlled Disease		Clinical Benefit	
Variables	HR (95% CI)	*p* Value	HR (95% CI)	*p* Value
Sex (male, female)	0.113 (−0.216, 0.441)	0.487	0.289 (−0.036, 0.614)	0.079
Histological variant (adenocarcinoma, squamous)	0.246 (−0.219, 0.712)	0.287	0.022 (−0.473, 0.518)	0.927
Previous lung surgery (yes, no)	0.036 (−0.314, 0.386)	0.835	−0.233 (−0.585, 0.119)	0.185
Line immunotherapy (first, ≥second)	−0.390 (−0.719, −0.060)	0.022	0.289 (−0.073, 0.651)	0.113
Drugs (pembrolizumab, nivolumab)	0.292 (−0.093, 0.678)	0.131	−0.100 (−0.518, 0.318)	0.627
SUV_maxTL_ (<11.4 vs. >11.4)	−0.072 (−0.475, 0.331)	0.717	-	-
TLG_WB_ (<194.1 vs. >194.1)	0.215 (−0.179, 0.610)	0.272	-	-
MTV_WB_ (<54 vs. >54)	0.215 (−0.179, 0.610)	0.272	-	-
SUV_peakTL_ (<9 vs. >9)	0.005 (−0.398, 0.408)	0.979	0.056 (−0.363, 0.475)	0.787
ΔSUV_maxTL_ (<0.3 vs. >0.3)	−0.359 (−0.736, 0.018)	0.061	-	-
ΔTLG_WB_ (<4.35 vs. >4.35)	−0.790 (−1.039, −0.541)	<0.001	0.622 (0.285, 0.960)	<0.001
ΔMTV_WB_ (<−2.55 vs. >−2.55)	−0.426 (−0.790, −0.061)	0.024	0.678 (0.359, 0.996)	<0.001
ΔSUV_peakTL_ (<−0.21 vs. >−0.21	−0.503 (−0.852, −0.153)	0.007	0.156 (−0.260, 0.572)	0.449

SUV: standardized uptake value; TLG: total lesion glycolysis; MTV: metabolic tumor volume; TL: target lesion; WB: whole-body.

## Data Availability

The data presented in this study are available on request from the corresponding author.
